# The effect of target scarcity on visual foraging

**DOI:** 10.1098/rsos.240060

**Published:** 2024-12-04

**Authors:** A. E. Hughes, H. R. Statham, A. D. F. Clarke

**Affiliations:** ^1^ Department of Psychology, University of Essex, Colchester, UK; ^2^ School of Psychology, Cardiff University, Cardiff, UK

**Keywords:** foraging, visual search, cognitive modelling

## Abstract

Previous studies have investigated the effect of target prevalence in combination with the effect of explicit target value on human visual foraging strategies, though the conclusions have been mixed. Some find that individuals have a bias towards high-value targets even when these targets are scarcer, while other studies find that this bias disappears when those targets are scarcer. In this study, we tested for a bias for scarce targets using standard feature versus conjunction visual foraging tasks, without an explicit value being given. Based on the idea of commodity theory and implicit value, we hypothesized that participants would show a scarcity bias. The bias was investigated using a Bayesian statistical model which has been developed for predicting target-by-target foraging behaviours. However, we found no evidence of a scarcity bias in our experiment, suggesting that participants did not inherently find rarer targets more rewarding.

## 1. Introduction

Humans and other animals search their environment regularly, looking both for unique items and for items where there are multiple exemplars available (such as berries on a bush). The former situation can be conceptualized as a visual search task where an observer must find a single target type from among distractors. The latter is referred to as foraging, in which observers search and collect multiple targets. It is considered an important behaviour given its connection to resource gathering [1] and has even been argued to be a key behaviour driving human cognitive evolution [2,3].

**Figure 1.**
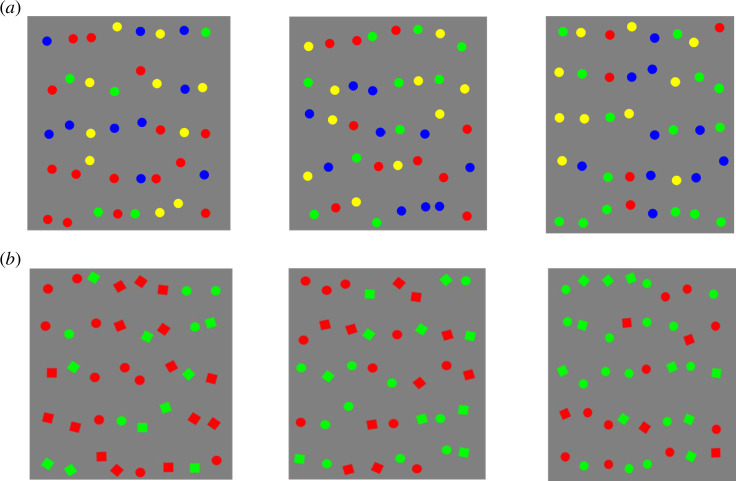
Example stimuli. (*a*) Stimuli for the feature search, (*b*) conjunction search. For feature, from left to right; is scarce green target condition, equal target condition, and scarce red target condition. For conjunction search, from left to right; scarce green circle condition, equal targets condition and scarce red squares condition.

A number of studies have addressed the factors that influence foraging behaviour. Early work by Dawkins [4] observed that chicks appear to peck grains in ‘runs’ of one-grain type before switching to the other. More recently [5], this behaviour has been studied in humans. When human participants are tasked with collecting multiple target types by sequentially tapping on them during an iPad game, they tend to consecutively select targets of one type in a series of ‘runs’. This is especially the case in relatively difficult ‘conjunction’ searches where the targets are defined by a combination of two features, compared with ‘feature’ searches where just one feature can be used to differentiate targets and distractors (see figure 1 for examples of the displays). This method appears to be more efficient compared with switching between target types [6].

Target value has been shown to be one of the main factors governing the deployment of visual attention [7], and as such, it plays an important role in foraging behaviour. Value can be thought of as either explicit or implicit. An example of explicit value influencing foraging behaviour can be seen in a study by Nityananda & Chittka [8]. They trained bees to discriminate between artificial flowers, their foraging behaviour was influenced such that they chose the more rewarding flowers (with more sucrose) in higher proportions. In humans, we can also use points and prizes as a way to manipulate explicit value, e.g. by tying participant payment to gaining a certain number of points in an experiment. By contrast, implicit value is used to describe situations in which some targets may be more attractive to a forager owing to an incidental feature. For example, Nityananda & Chittka [8] manipulated saliency in their experiments by adjusting the colour contrast of targets compared with their background and found that higher saliency targets were more likely to be selected than lower ones.

One interesting example of implicit value is *scarcity*, where a target type is visually less common in an environment. Brock’s Commodity Theory is a theory from social psychology which suggests that participants may value scarce targets (or other ‘useful things’, including messages and experiences as well as material objects [9]) more highly than readily available targets [10]. Empirical evidence for this theory has been found across a range of contexts [11] (although not all studies have found strong evidence for scarcity biases e.g. [12]). A preference for scarce objects could occur because possessing a scarce object may provide a feeling of personal distinctiveness or uniqueness [11], or because scarcity can be taken as a proxy for other desirable features, such as popularity. However, there may also be more ‘low-level’ explanations for a scarcity preference: for example, it has been argued that participants show stronger sustained attention to scarce resources, leading to more intense evaluation of the item [13], and therefore perhaps changing behaviour.

Both explicit target value and scarcity have been manipulated in the context of foraging studies. For example, Wiegand & Wolfe [14], manipulated explicit target value and prevalence (which can be considered equivalent to scarcity) and found that participants preferentially selected targets of higher value. However, this effect was modulated by target prevalence: participants no longer showed a preference for the higher value targets when they had a much lower prevalence than the lower value targets. In another study, Tagu & Kristjánsson [15] had participants collect a certain number of points by collecting targets, and the high-value targets were rarer than lower value ones. In contrast to Wiegand & Wolfe [14], participants tended to select high-value targets earlier than low-value targets, even though the high-value targets were scarcer. Wolfe *et al*. [16] investigated the effect of target prevalence in a hybrid foraging task where all four possible targets had equal value and found that participants picked the more common targets at a higher rate than the less common ones, an effect they attributed to priming. Thus, there have been mixed results in the human foraging literature on the effect of target scarcity on human foraging. It is worth noting that all the studies to date did explicitly assign targets a value (even if equal) and therefore do not necessarily tell us about the implicit value that participants may assign to targets.

There have been a number of visual search studies that have investigated scarcity, focussing on the context of scarce or abundant distractors rather than studying the effect of scarce targets. In one study, where participants searched for a target among two types of distractors, the distractor ratio strongly affected behaviour, with participants responding more quickly when one distractor was rarer than the other [17]. Similarly, people have been shown to be able to search relatively efficiently for a conjunction target within the smaller group of distractors when one type of distractor is more numerous than the other [18]. Attention is therefore directed to the rarer elements of the display, in a manner that might share cognitive similarities to a ‘scarcity bias’, although the authors themselves argued that this behaviour was observed because it is faster to start a search in a smaller group compared with a larger one.

In previous studies of foraging behaviour, differences between conditions have been studied using aggregate statistics, such as the number of ‘runs’ of a particular target type (i.e. the number of times that target is selected in a row) or the total number of targets found in the longest run. However, these measures have limitations. For example, they can be biased by the spatial layout of the display. They also do not allow us to distinguish between the case where a participant sticks with a particular target because of a preference for that specific target type, compared to the case where they simply like to stay searching for the same target template, regardless of what the target is. Similarly, with these aggregate statistics methods, it is not necessarily intuitive to account for imbalances in target numbers in a display, and how we can conclude whether a ‘scarcer’ target is selected more or less often than would be expected by chance: for example, if there are fewer of target A compared with target B, a reduced number of switches between target types compared to the case where the targets are equal in number may simply reflect the fact that fewer switches are possible.

To be able to more precisely track participant behaviour in these types of foraging tasks, we have developed a generative Bayesian model based on sampling without a replacement procedure. The benefit of this model is that it is able to break down behaviour into a series of cognitive biases, such as a preference for sticking with the same target type, or a preference for selecting a nearby target type, thus overcoming some of the limitations inherent to analyses based on aggregate statistics. We have successfully demonstrated that our model can account for average patterns of behaviour in a range of human foraging experiments [19]. In addition, it can make relatively accurate predictions of the next target a given individual will select on a trial [20]. We therefore think that our model is a powerful tool for studying and understanding the processes underlying human foraging behaviour.

In the current study, participants searched among coloured targets and distractors, in feature and conjunction styles of foraging like that of [5]. In the feature task, participants had to distinguish between targets and distractors based on colour. In the conjunction task, both shape and colour had to be considered in order to differentiate targets and distractors. Participants took part in some conditions where there were equal numbers of each target type, and other conditions where one target type was more numerous than the other. We had two key aims. First, we tested whether scarcity affects how participants forage. If participants implicitly value the scarcer target more highly, as Brock’s Commodity Theory [10] suggests they would, we expected participants to show a preference for the scarcer target, as measured by the bias parameter in the generative foraging model. As a secondary aim, we tested the extent to which the results of [19] generalize to a novel set of data. To date, we have used only secondary datasets, and therefore we used the registered report format to pre-register specific hypotheses relating to the parameters in our model to test how generalizable previous findings are.

## 2. Methods

### 2.1. Participants

We collected data from 36 participants, recruited from the University of Essex participant pool. This sample size is justified below in §4.4. Informed consent was collected at the beginning of the study and the participants were debriefed as to the nature of the study afterwards. Participants confirmed (via self-report) that they had normal or corrected-to-normal vision. Ethical approval for the experiment was granted by the University of Essex Research Ethics Sub-committee 1 (ETH2223−1093). The experiment took around 30 min to complete, and participants were compensated £5 for their time.

### 2.2. Design

A 2 × 3 within-subjects design was used. The first independent variable was the difficulty of the task, with two levels: *feature* and *conjunction* (details of the stimulus manipulation given in the following sections). The second independent variable was the ratio of targets of class A to B, with three levels: target class A is *scarce* (5 A to 15 B); class B is *scarce* (15 A to 5 B) and an *even* ratio (10 A to 10 B).

Each participant participated in all six conditions. Within each condition, there were 10 trials, meaning that they completed 60 trials. Each condition was completed as one block, and the order of blocks was counterbalanced across participants (half the participants completed the three feature blocks first, in a random order, and half completed the three conjunction blocks first, in a random order).

Before beginning the experiment, each participant completed a practice trial to familiarize themselves with the procedure: this was similar to a *feature* trial with an *even* ratio of targets but using different colours (black and white for the targets, and ‘old lace’ and ‘thistle’ for the distractors: all colour names refer to the red, green, blue hex colours) and shapes (all targets and distractors were triangles, approx. 1 unit of visual angle wide). All other features otherwise exactly resembled the main experimental trials.

### 2.3. Stimuli

The experiment was created in PsychoPy-2022.2.4 software, and the code is available in our GitHub repository. Each trial consisted of 40 items on a grey background. These items were organized in a grid but their placement was slightly jittered to create some irregularity, following previous studies [5,21]. Each trial included 20 targets and 20 distractors, and their position was randomized. In the feature task, blue and yellow circles were the distractors while green (‘lime’ was used as the green colour in all cases), and red circles were the targets. In the conjunction task, red circles and green squares were the distractors while red squares and green circles were the targets. Circle targets and distractors had a radius of 25 pixels (approx. 1 unit of visual angle), and square targets and distractors had a width and height of 25 pixels (as before, approx. 1 unit of visual angle). Examples of the stimuli can be seen in figure 1.

### 2.4. Procedure

Participants completed the experiment in a quiet room with normal illumination. The experiment was conducted on a Dell Optiplex 7050 computer, with a screen size of 1920 × 1080 pixels (though the targets were placed within a grid of 1000 × 1000 pixels in the centre of the screen). Participants sat with their head stabilized using a chin rest at a distance of 60 cm from the screen. Participants began each block by reading instructions telling them which items were the targets for that block. In each trial, they ‘collected’ items by clicking on them using a computer mouse. Once a target item was clicked, it immediately disappeared from the screen. If a participant clicked on a distractor, the trial immediately ended and it was restarted (up to a maximum of five attempts per trial): this was done to follow the procedure used in previous studies [5,21]. Trials ended when the participant had clicked on all targets, again following previous works [5,21]. Participants had to complete five valid trials with no mistakes in each condition.

Participants had their eyes tracked for the duration of the experiment using an SR EyeLink 1000 Plus eyetracker. These data are available for future exploratory analysis but are not used in the current registered report.

## 3. Data analysis

We used the four-parameter generative foraging model proposed by [19] to analyse the data. The model allows for target-by-target prediction of behaviour during visual foraging [20] and a benefit of this approach is that it enables us to parameterize the factors that may affect the forager’s choice of targets, such as proximity or a preference for foraging in ‘runs’ of a single target type [5]. The model also contains a ‘class bias’ parameter which detects a preference for one target type over another type. If item scarcity does make some targets more attractive than others, we should be able to see a difference in this parameter between the *scarce* and *even* conditions.

We have previously shown that the model can detect differences in this parameter between value and no-value conditions of previously collected data [15,19], and provide justification in §4.4 where we believe that we can detect small differences in target preference in our experimental design.

### 3.1. A four-parameter model of visual foraging

We model foraging as a process of weighted sampling without replacement. We assume that target items belong to one of two classes (A and B) and details of any distractor items are neglected. In the experiment presented in this manuscript, A and B are red and green circles, respectively, for the feature task, then red squares and green circles for the conjunction task.

The probabilities of each remaining target items are updated after each selection depending on four parameters defined as follows:

- $b_{A}=\text{logit}(p_{A})$: the logarithm of the odds of $p_{A}$. $p_{A}$ can be thought of as the probability of selecting an item of class A compared with class B, all else being equal. Similarly, $b_{B}=\text{logit}(p_{B})$ is the logarithm of the odds of relative attractiveness of B over A. This attractiveness could be owing to properties such as low-level salience or reward and value. A value of $b_{A}=0(p_{A}=0.5)$ corresponds to a situation in which items from both classes are equally likely to be selected next. The further away this parameter is from zero, the stronger the preference for A over B is. In this experiment, we predicted that $b_{A}$ would be more strongly positive in the case where A is the scarce target category and would be more strongly negative when B is the scarce target category;
- $b_{S}=\text{logit}(p_{S})$: the logarithm of the odds of $p_{S}$, the preference for selecting an item of the same class as the previously selected item. High values of this parameter lead to ‘sticky’ behaviour with long runs of the same item class, while low values lead to switching behaviour in which participants alternate which item class they select. $b_{S}\approx 0$ indicates that the class of the previously selected item has little effect on which item will be selected next;
- $\sigma_{\rho }$: this parameter reflects the importance of proximity when selecting the next item. The larger $\sigma_{\rho }$ is, the more heavily weighted the selection is to items that are close to the previously selected item; and
- $\sigma_{d}$: measures relative direction. The larger this parameter is, the larger the preference there is for selecting items that are ‘ahead’ of the previously selected item. As this parameter becomes more negative, this behaviour flips and there is a preference for selecting items ‘behind’.

These four parameters are fitted to the data for each experimental condition: $b_{A}(k),b_{S}(k),\sigma_{\rho }(k)$ and $\sigma_{d}(k)$ where k is one of K experimental conditions. Further details of the model implementation can be found in the electronic supplementary material.

### 3.2. Implementation details

The model fitting procedure was the same as Clarke *et al*. [19] with three changes. Firstly, while a multi-level framework was used to account for the differences between participants, we did not model the correlations between random effects. This is because we previously found relatively weak correlations when modelling similar experiments in Clarke *et al*. [19], and this modification significantly reduces computational time. Secondly, we adjusted the manner in which relative distances are calculated in order to account for the fact that the stimulus display is not necessarily square (although we used a square display in this experiment).^1^

The following weakly informative priors were used:^2^


(3.1) $$b_{A},b_{B}∼N(0,1),$$

(3.2) $$b_{S}∼N(0,1),$$

(3.3) $$\sigma_{p}∼N(15,5),$$

(3.4) $$\sigma_{\rho }∼N(0,1).$$

Each prior was a normal distribution with a specified mean and s.d., and the values chosen were based on applying the model to data from previous related experiments [19]. Importantly, we used the same set of priors for both the *equal* and *scarce* experimental conditions.

Models were fitted using R [22] and Stan [23] (full details of the software environment are included in the electronic supplementary material). The model fit was checked to ensure that $\hat{r}<1.01$ and the trace plots were visually inspected to check convergence.

Applying sophisticated modelling to new data can sometimes lead to unexpected problems. Such issues can sometimes be easily solved by using a different set of priors, or some other change to how the model is implemented. We have aimed to transparently document all changes from our original plan by adding footnotes into the main manuscript where relevant.

Analysis materials are available on our GitHub repository and have been archived within a Zenodo repository.

### 3.3. Data exclusion

The following criteria were used for data inclusion/exclusion:

- data from terminated trials (owing to selecting a distractor) were not analysed; and
- any trial containing an inter-target selection time of more than 5 s was removed.

We only analysed data from participants who had at least five trials of data for each condition after the above criteria were applied. We collected enough data to ensure we had 36 participants for the final analyses after data exclusion criteria were applied.

## 4. Hypotheses

### 4.1. The effect of scarcity

Our main hypothesis (H1) was that participants will show a preference for selecting scarce targets. As preferences to select one target class over another may also differ owing to visual salience, we took $b_{A}($*equal*) as our baseline condition and compared this to $b_{A}({\text{scarce}}_{A})$ and $b_{A}({\text{scarce}}_{B})$.

We tested this hypothesis by examining the posterior distributions (given the data D) for the difference between these parameters: if both


(4.1) $$\begin{array}{ccc} & Pr(b_{A}({\text{𝑠𝑐𝑎𝑟𝑐𝑒}}_{A})-b_{A}(\text{𝑒𝑞𝑢𝑎𝑙})>0|D)>0.99 & \end{array}$$

and


(4.2) $$\begin{array}{ccc} & Pr(b_{A}(\text{𝑒𝑞𝑢𝑎𝑙})-b_{A}({\text{𝑠𝑐𝑎𝑟𝑐𝑒}}_{B})>0|D)>0.99 & \end{array}$$

are true, marginalizing over the *feature* and *conjunction* conditions, then we can conclude in favour of our hypothesis. We also used the same procedure to measure the effect of scarcity in the *feature* and *conjunction* conditions separately, although we had no specific hypothesis about the size or direction of potential effects.

In stage 1, we indicated that if we did not find strong evidence in favour of our hypothesis, we would carry out exploratory analysis to (i) investigate if one of the counterbalanced conditions showed a scarcity effect but not the other and/or (ii) investigate the extent to which it holds in a subset of participants. If there was a range of scarcity effects across different participants, we said we would explore whether these were correlated with the other parameters in our model.

We also said that in the unlikely event that we were unable to achieve a good model fit using the full four-parameter model, we would fit a simpler ‘sampling without replacement’ model which ignores the spatial components of the model [19]. However, this was not necessary.

### 4.2. Secondary hypotheses around model fit

We also tested a number of secondary hypotheses to test the extent to which the results of [19] generalize to a novel set of data:

H2: if our *feature* versus *conjunction* manipulation showed a similar effect as that seen in [5,21], we expected to see a larger value for $b_{S}$ in the *conjunction* condition compared with the *feature* condition. This was investigated by examining the posterior distribution for a difference between feature and conjunction conditions using the same procedure as above.

H3: we predicted there will be a large ($\sigma_{\rho }>10$) proximity bias in both conditions. Previous work has shown values of around $\sigma_{\rho }=20$ are typical. We expected the effect of proximity to be larger in the *feature* condition, based on findings from [5] and [21] (as analysed in [19]). This was investigated in a similar manner to [H2].

H4: we predicted we will see a negative effect of relative direction, although we predicted this effect would probably be weak (around −1) with considerable variation between individuals. In order to test this hypothesis, we calculated whether 99% of the posterior distribution for the relative direction parameter was negative.

### 4.3. Planned exploratory analyses

A new version of our model was released by the time we collected our data, so we also present results using this (in the electronic supplementary material). This model does include the full random effects correlation structure. An LKJ prior was used for the random effect structure [24].^3^

We also present (in the electronic supplementary material) the standard aggregate descriptive statistics used in foraging research (maximum run length and total number of runs). We do not use these in our analyses but they provide a useful reference point for comparisons with previous research in the field.

### 4.4. Justification of sample size

We used a simulation approach to justify our sample size. In short, we used our generative model of visual foraging to simulate data for a given set of parameters. Based on the results from [19], we set $b_{S}=1$ (i.e. $p_{S}=0.73$) for the feature condition with $\sigma_{\rho }=15$ and $\sigma_{d}=-1$. For the conjunction condition, we set $b_{S}=2$, $\sigma_{\rho }=10$ and $\sigma_{d}=-1$. For the equal condition (in both the feature and conjunction cases), we made both target types equally likely ($b_{A}=0$) while in the scarce condition (again, for both feature and conjunction), we assumed a small preference for the less common target type of $p_{A}=0.6$ (i.e. $b_{A}=0.405$). This effect was chosen so that it was somewhat smaller than the effect of explicit value found in data from [15] of 0.75. It is also similar to the target preference seen in [21]: in this experiment, there was no specific experimental manipulation or hypothesis regarding a bias for one target or another, so we would expect this to be a reasonable minimal effect size of interest.

**Figure 2.**
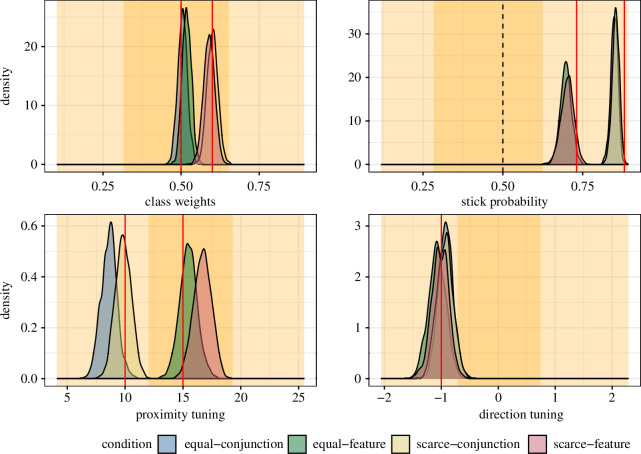
Posterior probabilities after fitting the model to the simulated data. The yellow-shaded background areas represent the prior distribution (53% and 97% Highest Posterior Density Intervals). Red lines indicate the parameter values used in the simulation.

We simulated 36 participants, each completing five trials per condition (this was a highly conservative estimate: in most cases, we expected that each participant will complete 10 trials per condition). While five trials may seem a relatively small amount, we have shown previously that good parameter estimates can be recovered with as little as one trial of data in a similar task [19]. The parameters for the random effect structure were again based on results from [19] (see the electronic supplementary materials for full details and code). We then fitted the four-parameter foraging model to these simulated data, the results of which can be seen in figure 2. We can clearly detect the bias towards the less common target type in scarce conditions. We can also see clear differences between the feature and conjunction conditions in stick probability and proximity tuning, demonstrating that we should have been able to detect these effects if they were present in the data.

### 4.5. Pilot results

**Figure 3.**
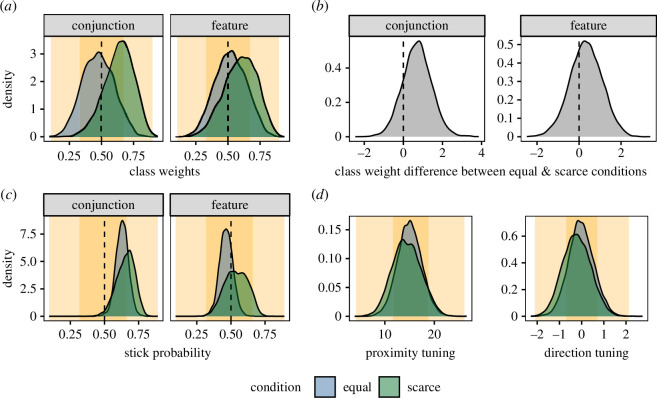
Posterior probabilities after fitting the model to the pilot data. The orange-shaded background areas represent the prior distribution (53% and 97% HPDIs). (*a*) Posterior distribution for $p_{A}$. (*b*) Posterior distributions for the difference between the *scare* and *equal* conditions. (*c*,*d*) Posterior distributions for the other model parameters.

Pilot data was taken from three female participants, each performing five trials in all six conditions. When applying [19]’s model, we see in the $p_{S}$ parameter a replication of Kristjánsson & Jóhannesson’s [5] findings; participants tended to stick to one target type more in the conjunction tasks compared with the feature tasks. In the $p_{A}$ parameter, the is a slight preference for scarce targets in the conjunction task (see figure 3). We can also see a proximity bias of approximately the expected size, although no real suggestion of a directional bias (although previously we have found this is a rather small effect). Overall, the pilot data showed that in a small number of participants, the effects seen were generally in line with our hypotheses.

## 5. Results

### 5.1. Data preparation and demographics

We collected data from 43 participants but following the removal of participants where there were technical faults or the experiment was not completed (see the electronic supplementary material for full details), we used data from 36 participants in our final analyses. We applied the data exclusion criteria outlined above, and removed 55 trials which contained an inter-target selection time of more than 5 s, but all participants continued to have at least five trials per condition. Participants had a mean age of 23.9 (s.d. = 4.2) and 26 of our participants identified as female.

All data and digital materials/code can be found in our GitHub repository. Note that each participant data file contains a date stamp, and we save the experimental parameters (e.g. conditions and blocks run) with each set of participant data, to act as a laboratory log. The approved stage 1 protocol is available on OSF. The experiment was executed and analysed in the manner originally approved: any unforeseen changes in those approved methods and analyses have been noted in the following sections.

### 5.2. The effect of scarcity

**Figure 4.**
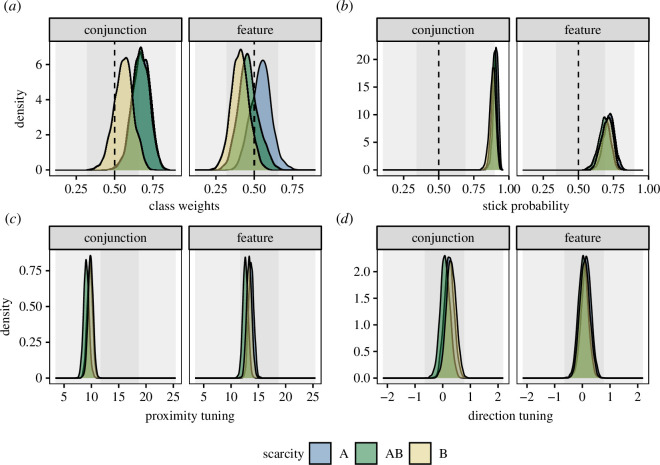
Posterior probabilities after fitting the model to the real data. The grey-shaded background areas represent the prior distribution (53% and 97% HPDIs). (*a*) Posterior distribution for $p_{A}$. (*b*) Posterior distributions for the stick probability $p_{S}$. (*c*) Posterior distributions for the proximity tuning parameter $\sigma_{\rho }$. (*d*) Posterior distributions for the direction tuning parameter.

We fitted the model as described in §3.2. Model checking procedures are detailed in the electronic supplementary material, but we assessed the model fit to be good based on standard procedures (e.g. checking traceplots visually for convergence). The posterior density distributions are illustrated in figure 4. Overall, we can see that many parameters have been estimated by the model to be very similar across the different conditions of the experiment (the posterior distributions overlap in many cases).

We hypothesized that participants will show a preference for scarce targets across both *feature* and *conjunction* conditions. Figure 4*a* shows the posterior distributions for $p_{A}$ i.e. the relative attractiveness of target A over target B. A posterior distribution shifted to the right of 0.5 indicates that target A was relatively preferred; a posterior distribution shifted towards the left of 0.5 indicates that target A was not preferred, and instead participants preferred target B.

In the *feature* condition, scarcity condition A (where target type A was less common) is slightly shifted to the right, and scarcity condition B (where target type B was less common) is slightly shifted to the left, as predicted. However, these are not very strong shifts. A similar pattern is seen in the *conjunction* condition, although there is a shift even for the AB condition (where the number of each target type was equal). This indicates that that target type A was preferred generally, for a reason other than scarcity.

Despite the slight trends seen in the graph, we did not find evidence for an effect of scarcity according to our pre-registered criteria: we did not find that 99% of the difference between the posterior distributions for the $b_{A}$ parameter was greater than zero for the two relevant comparisons, instead finding 67% for condition AB subtracted from condition A, and 82% for condition B subtracted from condition AB.

We also measured the effects of scarcity separately in the *feature* and *conjunction* conditions separately. Again, we did not find evidence for a scarcity effect based on the 99% criterion (see the electronic supplementary material for full details).

#### 5.2.1. Exploratory analyses for scarcity

Given that we did not find strong evidence in favour of our hypothesis, we carried out exploratory analyses. First, we investigated whether one of the counterbalanced conditions showed a scarcity effect but not the other (some participants completed *feature* conditions first, whereas others completed *conjunction* conditions first.^4^ There was no evidence for this suggestion, with neither group showing a scarcity effect (see the electronic supplementary material for full details).

**Figure 5.**
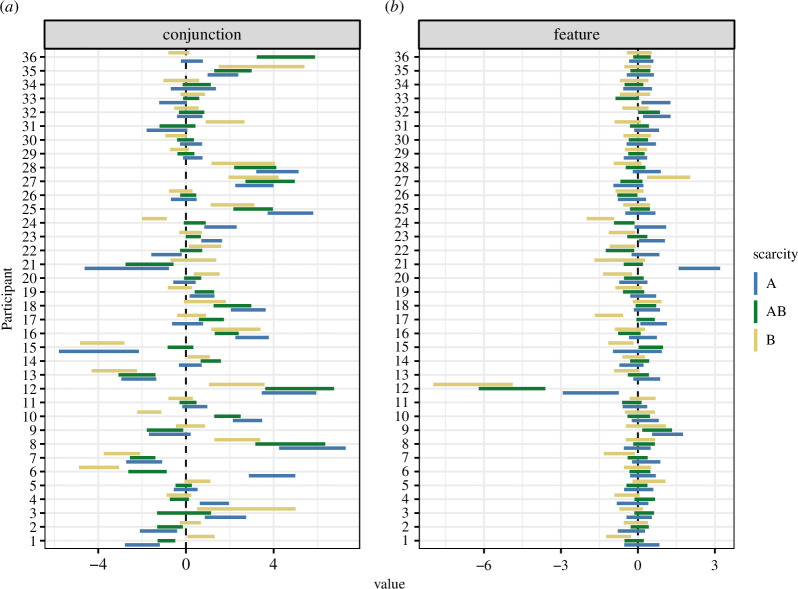
The random effects $u_{A}$ for each participant, for all scarcity and difficulty ((*a*) conjunction versus (*b*) feature) conditions.

Second, we investigated whether there was a subgroup of participants who showed a scarcity effect. From figure 5, we can see that participants tended to be fairly similar in their behaviour in the *feature* condition while there were a wider variety of strategies evident in the *conjunction* condition. Marginalizing across both *feature* and *conjunction* conditions, we did not see any individuals with a scarcity bias. However, if we consider just the conjunction condition, participant 6 shows evidence for a scarcity bias, and three other participants (10, 24 and 25) are close to meeting the evidence threshold. However, there is also one person (participant 1) who shows an anti-scarcity bias, being more likely to pick the most common target in each condition. It therefore seems likely that participants pick idiosyncratic strategies to complete the task in the *conjunction* condition: in some cases, this can look like a scarcity bias, but other strategies are also possible.

While there are a range of scarcity effects across different participants, we did not find good evidence that these are correlated with other parameters in our model (see the electronic supplementary materials for full details).

### 5.3. Secondary hypotheses around model fit

H2: we found a larger value for $b_{S}$ in the *conjunction* condition compared with the *feature* condition, as predicted (see figure 4*b*).

H3: we found a larger proximity bias in the *feature* condition compared to the conjunction condition, as predicted (see figure 4*c*), supporting H3. In both cases, the proximity bias was relatively large, although our original prediction that ($\sigma_{\rho }>10$) was only true for the *feature* condition.

H4: we did not find that 99% of the posterior distribution for the relative direction parameter was negative (see figure 4*d*). Therefore, our data does not support H4, where we predicted that we would see a negative effect of relative direction.

### 5.4. Planned exploratory analyses

In the electronic supplementary material, we present the standard aggregate descriptive statistics used in foraging research (maximum run length and total number of runs). In keeping with previous findings, we see that the *conjunction* conditions have a greater maximum run length and a smaller number of runs compared with the *feature* conditions. The model’s random effect parameters for each person for sticking with the same target type correlate well with their total number of switches, confirming that this parameter in our model maps well on to this traditional measure.

We also present results (in the electronic supplementary material) using a more recent version of our foraging model, which is mathematically equivalent to the main version used in this paper, but with a tidier code base. The basic findings remained identical to those presented in figure 4.

### 5.5. Further exploratory analyses

During the review process, a reviewer suggested that one reason for the lack of scarcity effects is that participants may need time to learn which target is scarcer, and therefore we might only see strong effects in later trials. We tested this by analysing the first half of trials separately from the second half (see the electronic supplementary material for full details) but we did not see any difference in the results in the first half versus the second half, and in both cases, there was no evidence for a scarcity bias.

## 6. Discussion

In the current study, we tested whether target scarcity affects how people forage. We predicted that participants would implicitly value the scarcer target in a display more highly, as suggested by Brock’s Commodity Theory [10]. However, our analyses suggested that there was no strong evidence for an effect of scarcity, either for group-level analysis or at the level of individual participants.

These results are perhaps surprising in the context of previous findings [9,11] which have suggested that scarcity effects can be found across a wide variety of contexts. However, it is worth noting that these studies predominantly relate to ’higher-level’ reasoning tasks rather than low-level perceptual ones: for example, in one study, participants were asked whether they preferred a scarce or available art print [25]. Our findings therefore perhaps support the idea that if people do have a preference for scarce objects, this may be a ‘cognitive’ bias that reflects high-level desirable factors, such as believing that items are scarce because they are popular, rather than a ‘low-level’ bias where people show stronger sustained attention to less common items [13]. Interestingly, a previous study showed that children and adults care more about variety than scarcity when selecting novel items, but were more likely to select scarce items for themselves when they were in a context with multiple recipients [12]. This perhaps strengthens the argument that the social context may be key to triggering scarcity effects.

A related, but distinct, idea is that the instructions given to the participants in the experiment may be important. In many previous social psychology studies on scarcity, the participants are explicitly told by the experimenter that one type of target is scarce, and another is more common, as in the example of the art print given above [25]. This is distinct from the current experiment, where participants are not told which stimulus is scarcer. However, prevalence studies in visual search have shown that these different scenarios may have different effects on search behaviour, with top-down instructions potentially altering low-level attentional biases [26]. This could suggest that while the scarcity effects may need a ‘top-down’ input that was not present in our experiment, they could still act via ‘low-level’ biases. An interesting avenue for future exploration could be to run an experiment where participants are specifically instructed that certain targets are more prevalent than others, and to see if this alters behaviour.

A final explanation is that perhaps our participants did find the scarcer targets more rewarding, but there were other factors in the experimental design that changed the ‘selection balance’, meaning that the participant behaviour on our task was mostly impacted by other, stronger factors [15]. Previous research using more cognitive tasks have found effects of target prevalence: in the most directly comparable study, where all targets had equal value, the effect was in the opposite of our prediction, in that participants were more likely to choose the more common targets [16]. We did not find any evidence for this type of ‘anti-scarcity’ bias in our study either. We think this may reflect some interesting methodological differences that perhaps altered the ‘selection balance‘. First, Wolfe *et al*. [16] used a design where participants were able to move on from a ‘patch’ at any time of their choosing, rather than the exhaustive search that our participants were required to complete. They also used four targets in their study, compared with our two, and targets made up a smaller proportion of the display (around 20−30%) than in our experiment. Finally, each target collected the participant points, and therefore participants aimed to collect as many points as possible over the experiment. It therefore seems likely that a sensible strategy for participants to maximize their points in their experiment would be to search for the most numerous targets, as these were still relatively rare and difficult to find in the display, and then move on to the next screen rather than spending a lot of time trying to find even rarer scarce targets. In our study, participants gained no explicit benefit from finding one type of target before the other, and our results suggest that there was no implicit benefit to them prioritizing either the more or less-frequent target type.

Despite finding no evidence for scarcity biases, we did see that participants had an overall preference for target type A in the *conjunction* condition: all three scarcity conditions showed a positively skewed class weight distribution. This was not seen in the *feature* condition, where in the AB condition, people showed a similar preference for the two target types. For the *conjunction* condition, the bias for target type A (red squares) seems unlikely to relate to differences in salience of the colours chosen: the same colours (red and green) were used in the *feature* condition, and did not seem to lead to bias. We therefore speculate that this effect may reflect a difference in shape salience, perhaps because circles were used as targets in all trials, so the square stimulus was slightly more novel and salient overall. Another possible explanation could be that the rotation of the targets matters: the square targets are more ‘unique’ compared to the circle targets, which all continue to have the same orientation when rotated, which may make the squares more salient. Interestingly, previous work using the same stimuli also shows a similar bias when analysed using our model e.g. the datasets in Clarke *et al*. [21] and Kristjánsson *et al*. [5], as re-analysed in Clarke *et al*. [19]. The design of our experiment means that this ‘baseline’ preference is unlikely to matter for understanding the effects of scarcity, but it is worth noting that these types of underlying saliency preferences appear to be stable and repeatable across individuals and experiments, and therefore it may be interesting in future work to try to understand more fully why they occur.

A secondary aim of this experiment was to test the generalizability of the results from Clarke *et al*. [19]. To date, we have only tested our modelling framework on secondary datasets, so we wanted to see to what extent the previous findings replicate to a novel, pre-registered set of data. In the main experiment, the findings were very similar to previous work: we find that people are more likely to stick with the same target type in the *conjunction* condition compared to the *feature* condition, as has been found in [5,21] and many other foraging studies. We also find the values of $b_{S}$ our model converged on for this set of experimental data were very similar to those found in [19]. Similarly, we found that proximity was an important parameter in the model, and the effect was larger for the *feature* condition, as predicted. However, the absolute values for the effect of proximity were a little smaller than we originally predicted, particularly for the *conjunction* condition. This may be owing to slight model adjustments compared to our original paper [19], particularly in the way that relative distance is calculated, or may reflect methodological differences. We would recommend that inferences about proximity using the model are based on differences between conditions within an experiment, as these seem to be replicable.

We predicted that we would see a negative effect of relative direction, with participants being slightly more likely to ‘track back’ on themselves to pick up targets behind the last selected one than to continue on in a straight line. We made this prediction because we found this effect in our re-analysis of [21] in Clarke *et al*. [19]. However, in the current dataset, we found no evidence for any type of direction bias, with the posterior distributions being centred on zero. One possible difference with the current experiment was that the number of targets was smaller than used in previous experiments (20 versus 40), which may make it harder to detect differences in directional strategy. Future modelling work could carry out sensitivity analyses to investigate this further.

## 7. Conclusion

Overall, we find no evidence that target scarcity affects participant’s behaviour in a foraging task, challenging Brock’s Commodity Theory [10] that suggests that participants should implicitly value scarce targets more highly. However, we are able to replicate many of the results from [19], suggesting that the modelling findings from that study broadly generalize to a new set of data.

We would argue that our modelling approach is a powerful method to develop our understanding of the cognitive processes that underlie human foraging behaviour. Given that many visual search tasks involve repeated sampling of the environment, even if only one final correct target is present, this modelling approach may also generalize fruitfully to a wider range of search behaviours. The modelling framework is also flexible, meaning that other relevant cognitive parameters can be easily included (e.g. memory), again broadening the scope of the questions that could be answered using this approach.

## Data Availability

Data and code are available on GitHub [27]. Supplementary material is available online [28].

## Footnotes

^1^
The original stage 1 report also included the statement: 'Finally, we have incorporated the initial selection bias described in Clarke *et al*. [20] into the full model: this allows us to take into account a person’s preference for starting each trial in a particular region of the screen (e.g. the centre, or the top left hand corner)’. This turned out to be more difficult to model than anticipated, and was not critical for testing our hypotheses, so we decided not to include this feature in the models presented in the paper, but we leave this statement here for full transparency.

^2^
Note that we made some minor adjustments in the s.d. values for these priors compared with the stage 1 registered report in order to facilitate model fitting.

^3^
Please note that in stage 1, this sentence was mistakenly included in §3.2 but in the main model presented in this paper, we do not measure the correlation between random effects, and so this prior is not relevant in that case.

^4^
Note that owing to a minor coding error and uneven data exclusion, we did not have exactly equal numbers in each condition: 14 participants completed the feature condition first, and 22 participants completed the conjunction condition first.
